# The clinical manifestation and the influence of age and comorbidities on long-term chikungunya disease and health-related quality of life: a 60-month prospective cohort study in Curaçao

**DOI:** 10.1186/s12879-022-07922-1

**Published:** 2022-12-16

**Authors:** Churnalisa Doran, Izzy Gerstenbluth, Ashley Duits, Norediz Lourents, Yaskara Halabi, Johannes Burgerhof, Adriana Tami, Ajay Bailey

**Affiliations:** 1 Department of Medical Microbiology and Infection Prevention, University Medical Center Groningen, Hanzeplein 1, 9713 GZ Groningen, The Netherlands; 2 Curaçao Biomedical and Health Research Institute, Pater Eeuwensweg 36, Willemstad, Curaçao; 3 Epidemiology and Research Unit, Medical and Public Health Service Curaçao, Piscaderaweg 49, Willemstad, Curaçao; 4 Department of Epidemiology, University Medical Center Groningen, Hanzeplein 1, 9713 GZ Groningen, The Netherlands; 5 Department of Human Geography and Spatial Planning, University of Utrecht, Heidelberglaan 8, 3584 CS Utrecht, The Netherlands

**Keywords:** Chikungunya, Asian, Genotype, Chronic, Long-term, Rheumatic, Arthralgia, Health-related quality of life

## Abstract

**Background:**

Persistent rheumatic symptoms and its impact on health-related quality of life (QoL), induced by the Indian Ocean Lineage (IOL) chikungunya virus (CHIKV) genotype have been widely studied. In 2014, a major CHIKV outbreak of the Asian genotype occurred in Curaçao, after which we established a longitudinal cohort in 2015, to follow the long-term CHIKV sequalae. Currently, the long-term clinical manifestations and its impact on QoL induced by the Asian CHIKV genotype, followed prospectively through time, and the association of age and comorbidities with rheumatic symptoms persistence, 60 months (M60) after disease onset is unknown.

**Methods:**

The cohort of 304 laboratory confirmed patients were followed prospectively in time at 3–16 months (M3-16), 30 months (M30), and M60 after disease onset. Demographic and clinical characteristics, and the 36-item short-form survey (SF-36) QoL status were collected through questionnaires. At M60, QoL scores were compared to general population (CHIK-) norms.

**Results:**

A total of 169 (56%) patients participated (74.6% female, mean age 56.1 years) at all time points, 107 (63%) were classified as recovered and 62 (37%) as affected. The affected patients reported an increase in the prevalence of arthralgia (P .001) and arthralgia in the lower extremities (P < .001), at M30 compared to M3-16. At M60, in comparison to recovered patients, affected patients reported a higher prevalence of recurrent rheumatic symptoms of moderate to severe pain, irrespective of age and comorbidities, and a higher prevalence of non-rheumatic symptoms (P < .001). Arthralgia in the upper (odds ratio (OR): 4.79; confidence interval (CI): 2.01–11.44; P < .001) and lower (OR: 8.68; CI: 3.47–21.69; P < .001) extremities, and headache (OR: 3.85; CI: 1.40–10.54; P = .009) were associated with being affected. The SF-36 QoL scores of the recovered patients were less impaired over time compared to the QoL scores of the affected patients. At M60, the QoL scores of the recovered patients were comparable to the CHIK- QoL scores.

**Conclusions:**

Rheumatic and non-rheumatic symptoms, and QoL impairment may persist, 60 months following infection with the Asian CHIKV genotype, similar to the IOL genotype disease sequelae. Further research is needed to follow the clinical manifestations and QoL impact of each CHIKV genotype.

**Supplementary Information:**

The online version contains supplementary material available at 10.1186/s12879-022-07922-1.

## Background

Chikungunya is a re-emerging mosquito-borne viral disease caused by an arthritogenic alphavirus. There are defined chikungunya virus (CHIKV) genotypes: the West African, East-Central South Africa (ECSA), ECSA-diverged or Indian Ocean Lineage (IOL), and an Asian lineage [1]. The incubation period ranges from 1 to 12 days, with 3–25% of the cases being asymptomatic [2]. The acute clinical presentation is characterized by non-rheumatic and rheumatic symptoms including acute high fever, headache, rash, fatigue, myalgia, severe poly-arthralgia, and joint weakness and stiffness in the extremities, resembling rheumatoid arthritis (RA) [3–5]. While the acute symptoms generally resolve within 21 days [6], long-term disease with unpredictable relapses of rheumatic symptoms can occur for years, inducing subsequent psychological non-rheumatic symptoms, severely impacting both the physical and mental health-related quality of life (QoL) [7–11].

Although CHIKV has been studied for almost 70 years, our knowledge about the disease duration, long-term clinical manifestations, and reasons for persistency of rheumatic symptoms is incomplete. It has been suggested that the long-term persistency and disease severity may be due to age at the time of acute disease, underlying comorbidities [12, 13] such as cardiovascular diseases (CVD), rheumatic disorders, diabetes mellitus (DM), and asthma, and CHIKV genotype dependence [14–16], with the highest prevalence of rheumatic symptoms caused by the IOL genotype, followed by the Asian and ECSA CHIKV genotypes, respectively [1].

The first evidence of autochthonous transmission of CHIKV in the Caribbean region and the Americas was reported in December 2013, on the Caribbean island Saint-Martin [17]. Phylogenetical analyses identified the Asian CHIKV genotype as the cause of the outbreak, which was genetically related to CHIKV genotypes reported in Indonesia, China, and the Philippines [18]. The long-term clinical manifestations and its impact on QoL, induced by infection with the IOL CHIKV genotype have been well studied, especially in La Réunion [8, 19, 20]. In 2015, following a major CHIKV outbreak in Curaçao, we established a prospective chikungunya cohort to follow chikungunya disease sequalae over time, and described the long-term clinical manifestation and its QoL impact, 3–16 months (M3-16) [10] and 2.5 years (M30) [11] after disease onset, in the Caribbean region. To date, the long-term outcome in terms of clinical manifestations and its impact on QoL induced by infection with the Asian CHIKV genotype, 60 months (M60) after disease onset is unknown. The present study aimed to fill this gap.

This study builds on the two previous studies [10, 11] and aimed to assess the prevalence and clinical manifestations of rheumatic and non-rheumatic symptoms induced by the Asian CHIKV genotype trough time, to determine characteristics significantly associated with being long-term affected, and to evaluate the possible association of age and comorbidities with the persistence of rheumatic symptoms, M60 after disease onset, in Curaçao. We then focused on comparing the QoL of recovered and affected chikungunya patients with the QoL of individuals from the general population.

## Methods

### Setting

Curaçao is a constituent country within the Kingdom of the Netherlands, located in the southern Caribbean Sea [21]. In June-July 2014, a CHIKV outbreak of the Asian CHIKV genotype took place in Curaçao. Phylogenetic analyses found that the Asian CHIKV was related to isolates from the outbreaks in the Americas, including those in Saint-Martin, Brazil, and Mexico [22].

### Study design and population

The present longitudinal study is part of a prospective cohort of 304 patients age ≥ 18 years (at baseline), selected trough 14 health centres representative of the population of Curaçao, presenting with acute febrile symptoms and a positive laboratory confirmed serology, reverse transcription-polymerase chain reaction, or indirect fluorescent antibody test during the CHIKV outbreak [10]. The cohort was not genotyped for the infecting CHIKV lineage. During an epidemic, it is not imperative that all cases should be subjected to viral sequencing, instead data from representative populations should be collected for sequencing, to identify and trace the spread of circulating virus variants [23]. We have reported the clinical manifestation of long-term chikungunya disease and its QoL impact among the cohort at baseline (M3-16) [10] and first follow-up survey (M30) [11], and this second follow-up survey builds on these previous time points. In June 2019, the same cohort was contacted for participation in the second follow-up survey by telephone and/or house visits. If patients did not wish to participate or could not be reached after three failed contact attempts, they were excluded from the study.

#### Chikungunya non-cases recruitment

From July 2019 to February 2020, house visits in the general population were conducted to recruit one unmatched chikungunya non-cases (CHIK-) per household, using a randomized household sample from the Central Bureau for Statistics of Curaçao. Chikungunya non-cases were defined as individuals aged ≥ 23 years who self-declared not having had acute clinical symptoms during the CHIKV outbreak. This inclusion criterium was chosen based on a previous publication, in which a 99% concordance between self-declared negative CHIK- status and a CHIKV negative serological test was reported [8].

### Data collection

After consent, data were collected using piloted health survey questionnaires, administered face-to-face by trained interviewers, at home or work according to the preference of the patient or CHIK-, to minimize attrition bias. Patient’s data were collected on socio-demographic (comorbidities: pre-chikungunya infection (and present at baseline), and post chikungunya infection) and economic characteristics, and clinical manifestations of chikungunya disease at 3 time points, at M3-16 [10], M30 [11], and second follow-up from June 2019 to March 2020 (M60).

At M60, patients answering ‘yes’ to (recurrent or constant) rheumatic and non-rheumatic symptoms at the time of interview were considered symptomatic, to minimize recall bias. Rheumatic symptoms included arthralgia, joint weakness and/or myalgia. Recurrent was defined as relapsing symptoms with periods of relief and subsequent reoccurrence or as constant symptoms, without periods of relief. In addition, the pain intensity measured using a Visual Analogue Scale (VAS) and relapse frequency (since M30) of rheumatic symptoms were collected. The QoL was measured using the 36-item short-form survey (SF-36). The SF-36 evaluates the physical and mental aspects of QoL with eight domains, which are weighted to the physical components summary (PCS) and the mental component summary (MCS). Higher scores indicate better functioning [24, 25].

The data of the CHIK- was collected using a piloted health survey questionnaire, which included questions on socio-demographic and economic characteristics, and SF-36.

At M60, the primary outcome measures were the prevalence of rheumatic and non-rheumatic symptoms, determinants of being long-term affected, and QoL. Patients were classified as being recovered or affected based on their self-declaration. Patients were asked the question “How would you describe the effect chikungunya disease has on you at this moment?’’, the patients could answer with: (1) I have constant chikungunya disease related complaints; (2) I have recurrent chikungunya disease related complaints; (3) I have recurrent and constant chikungunya disease related complaints; (4) I do not have any chikungunya disease related complaints. Patients answering ‘’I do not have any chikungunya disease related complaints’’ were classified as recovered, if not, they were classified as affected.

### Data analyses

Univariate analyses with a Bonferroni post hoc analysis set at P ≤ .001 were performed to give crude associations between the recovered or affected classifications, and explanatory variables. Results were expressed as means ± standard deviations (SD) or medians with interquartile ranges (IQR) for continuous variables, and counts and frequencies (percentages) for categorical variables. The valid percentages were used, if data was missing. Matched categorical variables of the recovered or affected patients over time were compared by performing the Cochran’s Q or McNemar test, accordingly.

If significant, the McNemar test was performed as a post hoc test to the Cochran’s Q test. The categorical variables between the recovered and affected patients were compared by performing the Fisher’s exact test. The association of persistent rheumatic symptoms with age (at acute disease onset and at second follow-up) and (pre-existing and post-infection) comorbidities were analysed for affected patients.

The age categories were based on literature for comparison with other similar studies and were chosen as follows; 18–29 years, 30–44 years, 45–59 years, and ≥ 60 years [26–30]. The comorbidities were categorized into rheumatic disorders (rheumatoid arthritis, arthralgia, joint swelling, and/or joint weakness), CVD (cardiac and vascular diseases, including hypertension), asthma, and allergies (hay fever, eczema, food intolerance etc.). The explanatory variables of P ≤ .001 were fitted in a multivariate binary regression model with recovery as reference. A backwards step-wise selection was followed to exclude non-significant variables of P > .05, expressed as odds ratios (ORs) and 95% confidence interval (CI).

All categorical analyses were performed using a Bonferroni post hoc analysis set at P ≤ .001. The matched continues QoL variables of the recovered or affected patients over time were analyzed with the Friedman’s test. If significant, the Wilcoxon test was performed as a post hoc test to the Friedman’s test. The continuous QoL variables were compared between the recovered and affected patients, and CHIK- by performing the ANOVA, Mann-Withney U test, or Kruskal–Wallis test, as appropriate. Separate PCS and MCS analyses were performed based on gender, age at the time of interview, and comorbidities. If significant, the Mann-Withney U test was performed as a post hoc test to the Kruskal–Wallis test.

The minimum clinically important difference of the PCS and MCS was defined as 2.5–5-point change criterion for improvement, by calculating the score differences of the PCS and MCS domains [31]. In addition, the data from the CHIK- were used as SF-36 reference values, to compare the QoL scores between the chikungunya patients and CHIK-. All statistical analyses were performed with SPSS Data Entry Station (SPSS Inc., version 23.0, Chicago, Illinois).

### Patient consent statement

Ethics approval was obtained from the Medical Ethical Committee of the Saint Elisabeth Hospital in Curaçao (Reference number: 2015-002). Written informed consent was obtained from all study participants.

## Results

Among the cohort of 304 patients, 169 patients were followed at all time points of the longitudinal study and were included at M60 (response rate 56%) (Additional file 1). The mean (SD) duration since acute chikungunya disease was 58.5 (3.0) months, and ranged 50–75 months. From the 169 chikungunya patients, 107 (63%) were classified as recovered, and 62 (37%) as affected. From the general population 151 CHIK- consented to participate.

### Socio-demographic and economic characteristics

In terms of socio-demographic and economic characteristics, no statistically significant differences were observed between the recovered and affected patients (Table 1). The majority of the study population was female, with a female to male ratio of 2.93:1. Among the recovered and affected most patients were aged between 45 and 59 years, 43 (40.2%) and 26 (41.9%) respectively, at the time of acute disease. The socio-demographic and economic characteristics of the chikungunya patients compared to the CHIK- group are shown in Additional file 2.

**Table 1:** Characteristics of the recovered and affected patients, 60 months after disease onset (n = 169)

	Total	Recovered	Affected	P-value^a^
**Participants, no (%)**	169	107 (63.3)	62 (36.7)	
**Gender, no (%)**				.04
Female	126 (74.6)	74 (69.2)	52 (83.9)	
Male	43 (25.4)	33 (30.8)	10 (16.1)	
Sex ratio (male/female)	0.34	0.45	0.19	
**Age acute disease, years (%)**				.87
18–29	13 (7.7)	7 (6.5)	6 (9.7)	
30–44	43 (25.4)	28 (26.2)	15 (24.2)	
45–59	69 (40.8)	43 (40.2)	26 (41.9)	
≥ 60	44 (26.0)	29 (27.1)	15 (24.2)	
**Age current, years (%)**				.80
18–29	4 (2.4)	2 (1.9)	2 (3.2)	
30–44	31 (18.3)	20 (18.7)	11 (17.7)	
45–59	71 (42.0)	43 (40.2)	28 (45.2)	
≥ 60	63 (37.3)	42 (39.3)	21 (33.9)	
**Education, no (%)**				.85
Primary school or less	44 (26.0)	30 (28.0)	14 (22.6)	
Secondary school	24 (14.2)	15 (14.0)	9 (14.5)	
Intermediate vocational school	73 (43.2)	44 (41.1)	29 (46.8)	
University (of applied sciences)	28 (16.6)	18 (16.8)	10 (16.1)	
**Occupation, no (%)**				.80
Unemployed/homemaker/student	24 (14.2)	15 (14.0)	9 (14.5)	
Paid work	107 (63.3)	66 (61.7)	41 (66.1)	
Retired	38 (22.5)	26 (24.3)	12 (19.4)	
**Income, no (%)**^bc^				.46
0–1000 ANG	31 (20.4)	19 (19.6)	12 (21.8)	
1001–3000 ANG	70 (46.1)	47 (48.5)	23 (41.8)	
3001–5000 ANG	27 (17.8)	14 (14.4)	13 (23.6)	
≥ 5000 ANG	24 (15.8)	17 (17.5)	7 (12.7)	
**Pre-existing comorbidities, no (%)**				
Absence of comorbidities	73 (43.2)	46 (43.0)	27 (43.5)	1.000
Rheumatic disorders^d^	49 (29.0)	32 (29.9)	17 (27.4)	.86
Cardiovascular diseases^e^	51 (30.2)	29 (27.1)	22 (35.5)	.30
Diabetes mellitus	23 (13.6)	13 (12.1)	10 (16.1)	.49
Asthma	16 (9.5)	8 (7.5)	8 (12.9)	.28
Allergies^f^	19 (11.2)	11 (10.3)	8 (12.9)	.62
**Post-infection comorbidities, no (%)**				
Absence of comorbidities	137 (81.1)	84 (78.5)	53 (85.5)	.31
Rheumatic disorders^d^	7 (4.1)	3 (2.8)	4 (6.5)	.26
Cardiovascular diseases^e^	19 (11.2)	16 (15.0)	3 (4.8)	.05
Diabetes mellitus	1 (0.6)	1 (0.9)	0 (0.0)	1.000
Asthma	2 (1.2)	1 (0.9)	1 (1.6)	1.000
Allergies^f^	8 (4.7)	6 (5.6)	2 (3.2)	.71

^a^Groups were compared using the Fisher’s exact test, with Bonferroni multiple post hoc analysis, two-sided p-value corresponds to the comparison of the proportions between the recovered and affected groups; ^b^Antillian Guilder, 1 ANG = 0.55 United States Dollar; ^c^Total recovered group n = 97, total affected group n = 55; ^d^Rheumatic disorders includes rheumatoid arthritis, joint pain, swelling, and weakness; ^e^Cardiovascular diseases includes, myocardial infarction, hypertension, hypotension, and hypercholesterolemia; ^f^Allergies, includes hay fever, eczema, food intolerance and other

### Long-term clinical manifestations

#### Evolution of rheumatic and non-rheumatic symptoms over time

**Fig. 1:**
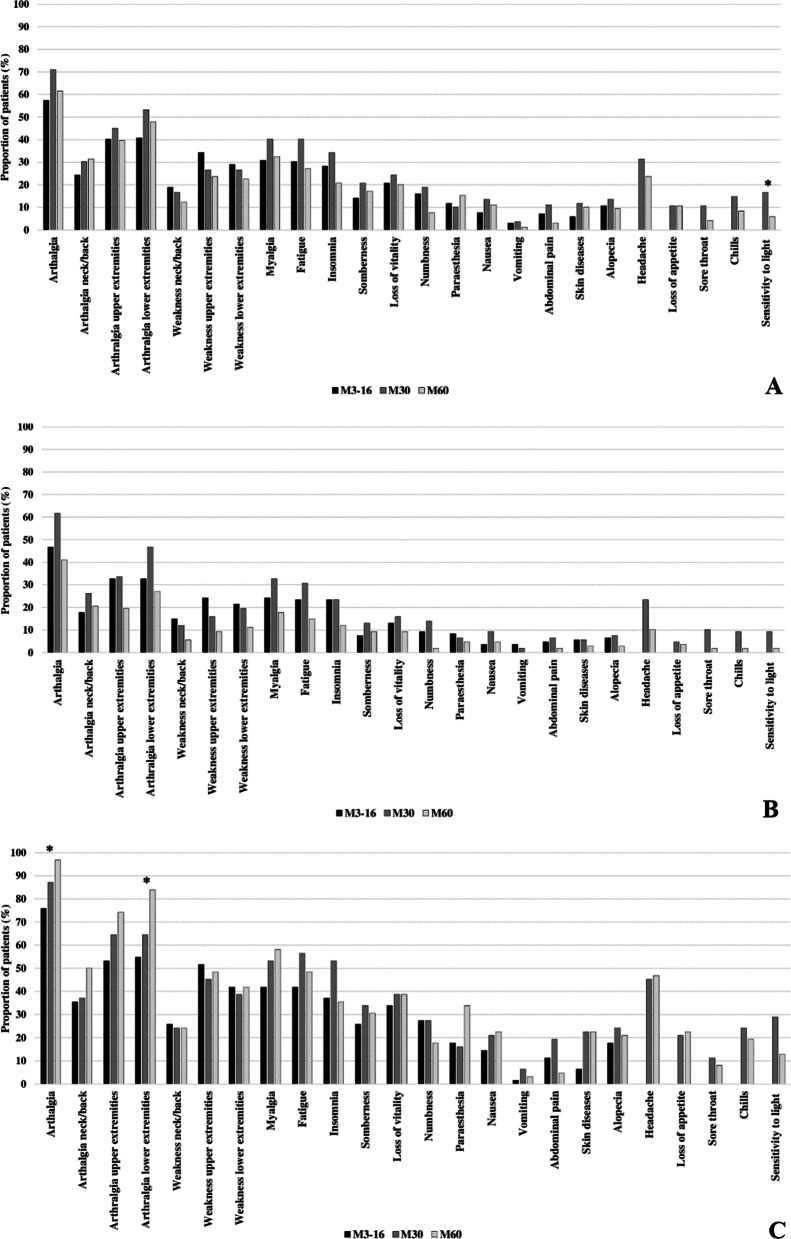
Evolution of rheumatic and non-rheumatic symptoms over time. Symptoms prevalence at baseline survey: 3–16 months after disease onset (M3-16), first follow-up survey: 30 months after disease onset (M30), and second follow-up survey: 60 months after disease onset (M60), were compared using the Cochran’s Q or McNemar test, with Bonferroni multiple post hoc analysis. The horizontal axis shows the rheumatic and non-rheumatic symptoms. Upper extremities refers to the shoulders, elbows, hands, wrists, and fingers; Lower extremities refers to the hips, knees, ankles, feet, and toes. Non-rheumatic symptoms measured since M30: headache, loss of appetite, sore throat, chills, and sensitivity to light. The vertical axis shows the proportion of symptomatic patients reporting ongoing symptoms, at the time of interviews. **A** Cohort (n = 169); **B** Recovered patients (n = 107); **C** Affected patients (n = 62). *Significant two-sided P-value (P ≤ .001) at M60 compared to M3-16

The cohort reported no significant difference in the prevalence of rheumatic and non-rheumatic symptoms over time, except for the non-rheumatic symptom sensitivity to light, which decreased significantly (P .001) at M60 compared to M30 (Fig. 1A).

Among the recovered patients, no significant difference in the prevalence of rheumatic and non-rheumatic symptoms were reported over time (Fig. 1B). Among the affected patients, no significant difference in the prevalence of rheumatic and non-rheumatic were reported over time, except for the rheumatic symptoms arthralgia (P .001) and arthralgia in the lower extremities (P < .001), which increased at M30 compared to M3-16 (Fig. 1C).

#### Prevalence and nature of symptoms at 60 months after disease onset

**Fig. 2:**
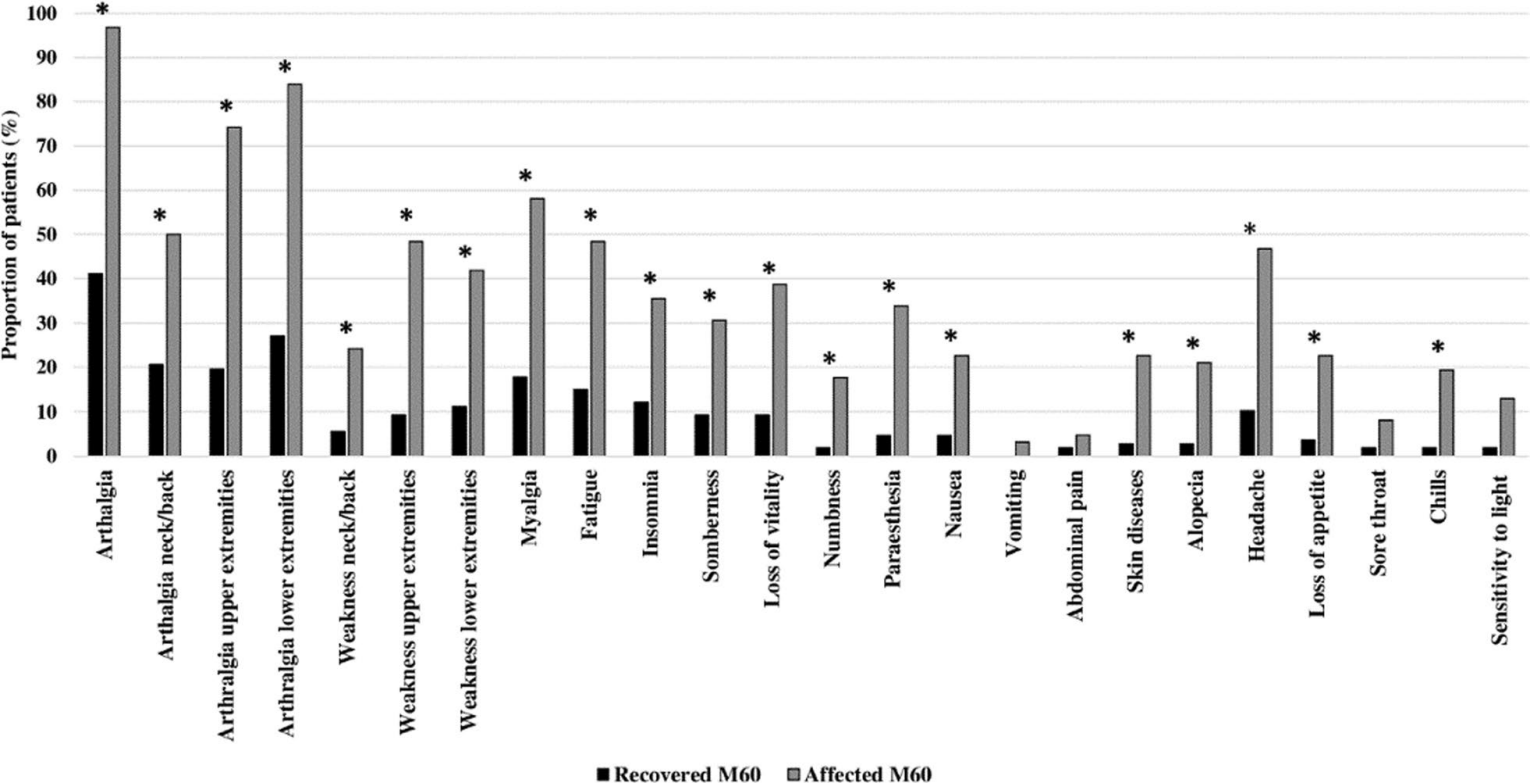
Rheumatic and non-rheumatics symptoms, 60 months after disease onset. Recovered (n = 107) and affected (n = 62) patients were compared using the Fisher’s exact test, with Bonferroni multiple post hoc analysis. The horizontal axis shows the rheumatic and non-rheumatic symptoms. Upper extremities refers to the shoulders, elbows, hands, wrists, and fingers; Lower extremities refers to the hips, knees, ankles, feet, and toes. The proportion of symptomatic patients reporting ongoing symptoms at the time of interview are shown in the vertical axis. *Significant two-sided P-value (P ≤ .001)

Among the affected patients, 60 (96.8%) reported experiencing arthralgia compared to 44 (41.1%) of the recovered patients (P < .001). Persistent rheumatic symptoms were significantly (P < .001) higher for affected patients compared to recovered patients. The most prominent persistent rheumatic symptoms were arthralgia in the upper and lower extremities, and myalgia, reported by 46 (74.2%), 52 (83.9%), and 36 (58.1%) of the affected patients, respectively (Fig. 2). The comparison between the recovered and affected patients at M3-16 and M30 are shown in Additional file 3.

In addition, most non-rheumatic symptoms were significantly (P < .001) more prevalent among the affected patients compared to recovered patients. Among the affected patients, 30 (48.4%) still experienced fatigue, 22 (35.5%) insomnia, and 24 (38.7%) loss of vitality. Among the non-rheumatic symptoms that were measured since (M30), the affected patients reported a significantly (P < .001) higher prevalence of headache 46.8% (n = 29), loss of appetite 22.6% (n = 14), and chills 19.4% (n = 12) compared to the recovered patients. Moreover, there was no significant difference in the constant or recurrent nature of symptoms between the recovered and affected patients (Additional file 4).

#### Pain intensity, relapse frequency and duration of rheumatic symptoms

The pain intensity and relapse frequency of rheumatic symptoms during the period between M30 and M60, was significantly (P < .001) higher among the affected compared to the recovered patients (Table 2). The experienced pain intensity was moderate (VAS 4–6) for 30 (48.4%), and severe (VAS 7–10) for 21 (33.9%) of the affected patients. The majority of affected patients (53.2%) reported 1–10 relapses, and 23 (37.1%) reported more than 20 relapses, with 48 (78.7%) reporting relapse duration of 1–6 days.

**Table 2:** Rheumatic symptoms pain intensity, relapse frequency and duration, reported by recovered and affected patients (n = 169)

	Recovered (n = 107)	Affected^a^ (n = 62)	P-value^b^
	n (%)	n (%)	
**Pain intensity (VAS score)**			** < .001**
None	79 (73.8)	0 (0.0)	
Mild (1–3)	13 (12.1)	11 (17.7)	
Moderate (4–6)	6 (5.5)	30 (48.4)	
Severe (7–10)	9 (8.4)	21 (33.9)	
**Relapse frequency**^c^			** < .001**
None	96 (89.7)	1 (1.6)	
1–10	10 (9.3)	33 (53.2)	
10–20	0 (0.0)	5 (8.1)	
> 20	1 (0.9)	23 (37.1)	
**Duration relapses**^d^			.68
1–6 days	9 (81.8)	48 (78.7)	
7–14 days	1 (9.1)	10 (16.4)	
15–21 days	0 (0.0)	1 (1.6)	
> 22 days	1 (9.1)	2 (3.3)	

^a^Total affected patients with recurrent symptoms n = 62; ^b^Groups were compared using the Fisher’s exact test, with Bonferroni multiple post hoc analysis, two-sided P-value corresponds to the comparison of the proportions of pain intensity, recurrent symptoms frequency and relapse duration (between first follow-up and second follow-up surveys, 30 and 60 months after disease onset), between the recovered and affected groups; ^c^Symptoms relapse frequency among recovered and affected patients, experiencing self-reported chikungunya related recurrent symptoms; ^d^Relapse duration of symptom among recovered and affected patients, experiencing self-reported chikungunya related recurrent symptoms. VAS = Visual Analogue Scale. Significant P-values after Bonferroni correction are indicated in bold

#### Association rheumatic symptoms with age and comorbidities

None of the persistent rheumatic symptoms differed significantly with age at acute disease onset (Table 3), nor increased age at second follow-up (Additional file 5).

**Table 3:** Persistent rheumatic symptoms of affected patients related to age at acute disease (n = 62)

	Age categories at acute disease (years)								
	Total	18–29		30–44		45–59		> 60	
	n = 62	n = 6		n = 15		n = 26		n = 15	
	n (%)	n (%)	P-value^a^	n (%)	P-value^a^	n (%)	P-value^a^	n (%)	P-value^a^
**Arthralgia in the**^b^									
Back/neck	31 (50.0)	2 (33.3)	.67	8 (53.3)	1.000	11 (42.3)	.44	10 (66.7)	.24
Upper extremities^c^	46 (74.2)	3 (50.0)	.17	14 (93.3)	.09	18 (69.2)	.56	11 (73.3)	1.000
Lower extremities^d^	52 (83.9)	6 (100)	.58	13 (86.7)	1.000	20 (76.9)	.30	13 (86.7)	1.000
**Weakness in the**^b^									
Back/neck	15 (24.2)	2 (33.3)	.63	5 (33.3)	.52	4 (15.4)	.23	4 (26.7)	.74
Upper extremities^c^	30 (48.4)	2 (33.3)	.67	9 (60.0)	.58	10 (38.5)	.21	9 (60.0)	.38
Lower extremities^d^	26 (41.9)	4 (66.7)	.23	7 (46.7)	.78	7 (26.9)	.07	8 (53.3)	.37
**Myalgia**	36 (58.1)	3 (50.0)	.69	10 (66.7)	.55	12 (46.2)	.13	11 (73.3)	.23

^a^Groups were compared using the Fisher’s exact test, with Bonferroni multiple post hoc analysis, two-sided P value corresponds to the comparison of the proportions of rheumatic symptoms and age categories among affected patients; ^b^Multiple answers were possible; ^c^Upper extremities refers to the shoulders, elbows, hands, wrists, and fingers; ^d^Lower extremities refers to the hips, knees, ankles, feet, and toes

In addition, none of the pre-existing or post-infection comorbidities were significantly associated with rheumatic symptoms (Additional files 6 and 7).

### Explanatory variables associated with being long-term affected, 60 months after disease onset

The socio-demographic characteristics and clinical symptoms with P ≤ .001, associated with being affected included in the binary logistic regression model, are shown in Table 1 and Table 2, and Fig. 2. In the multivariate binary analysis arthralgia in the upper (OR: 4.79; CI: 2.01–11.44; P < .001) and lower (OR: 8.68; CI: 3.47–21.69; P < .001) extremities, and headache (OR: 3.85; CI: 1.40–10.54; P = .009) were significantly associated with being affected by long-term chikungunya disease, 60 months after disease onset (Table 4).

**Table 4:** Final model of variables associated with being affected vs. recovered, 60 months after disease onset

	Odds Ratio (OR)	95% CI	P-value
**Arthralgia in the upper extremities**			
No	Reference	Reference	
Yes	4.79	2.01–11.44	< .001
**Arthralgia in the lower extremities**			
No	Reference	Reference	
Yes	8.68	3.47–21.69	< .001
**Headache**			
No	Reference	Reference	
Yes	3.85	1.40–10.54	.009

### Health-related quality of life scores

#### Health-related quality of life sores through time

**Fig. 3:**
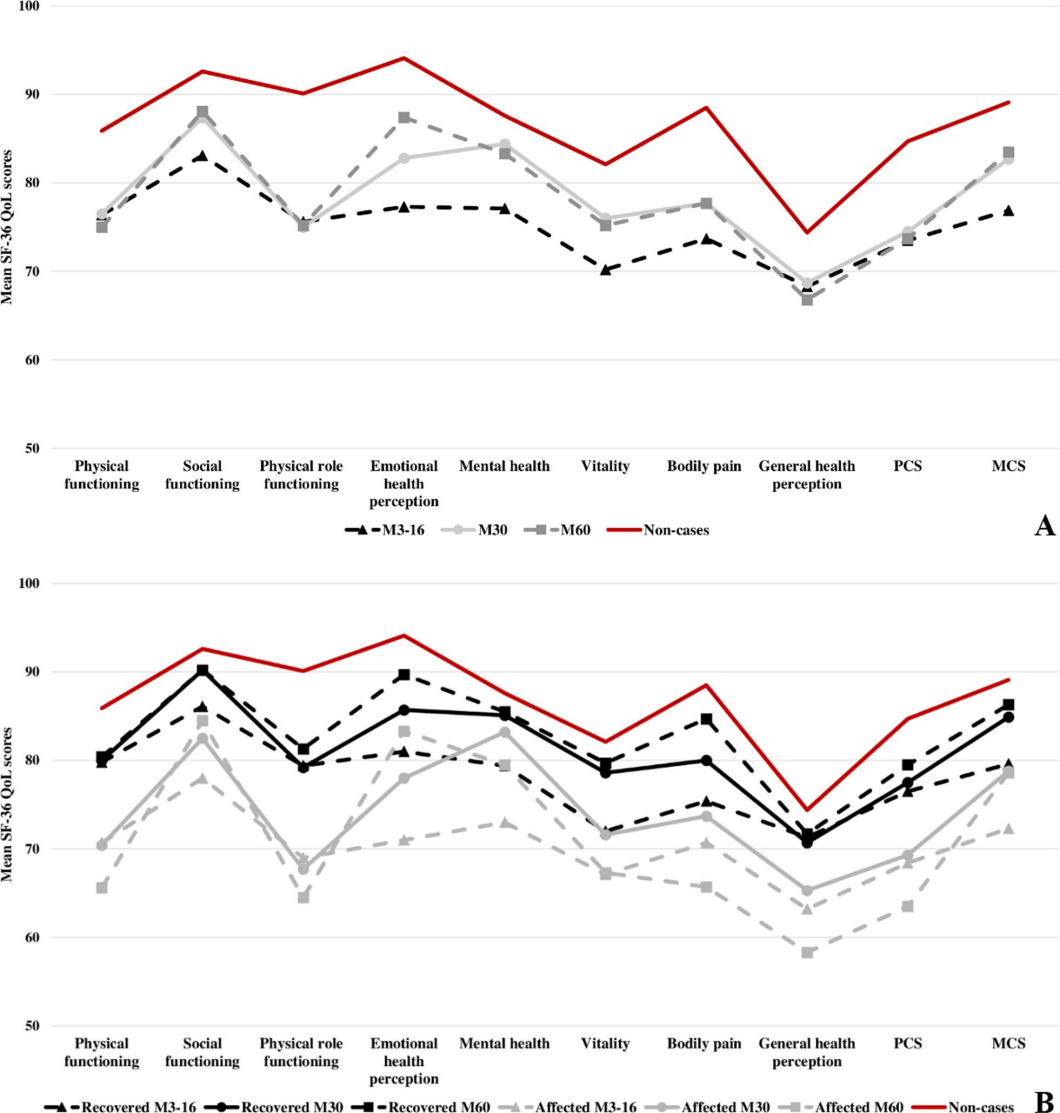
Mean 36-item short-form health survey quality of life (QoL) scores over time. **A** The QoL of the cohort (n = 169) in comparison to the QoL scores of the non-cases (n = 151); **B** The QoL scores of the recovered (n = 107) and affected (n = 62) patients in comparison to the QoL scores of the non-cases (n = 151). Physical component summary (PCS) includes the domains Physical functioning, Physical role functioning, Bodily pain, and General health perception. Mental component summary (MCS) includes the domains Social functioning, Emotional health perception, Mental health, and Vitality. M3-16 = baseline survey, 3–16 months after disease onset; M30 = first follow-up survey, 30 months after disease onset, M60 = second follow-up survey, 60 months after disease onset

The SF-36 QoL scores of the cohort were more impaired over time compared to the QoL scores of the CHIK-, at M60 (Fig. 3A). In addition, only the MCS scores of the cohort differed significantly (P < .001) over time, with an increase of 5.5 points at M30 compared to M3-16, and 4.6 points at M60 compared to M3-16 (Additional file 8). The SF-36 QoL scores of the recovered patients were less impaired over time compared to the scores of the affected patients. The QoL scores of the CHIK- were less impaired compared to the QoL scores of the recovered and affected patients, at M60 (Fig. 3B). The PCS scores of the recovered patients remained the same over time. The MCS scores of the recovered patients differed significantly over time, with an increase of 5.5 points (P .001) at M30 compared to M3-16, and an increase of 6.6 points (P < .001) at M60 compared to M3-16 (Additional file 9). The PCS and MCS scores of the affected were not significantly different and remained the same over time (Additional file 10).

#### Health-related quality of life sores at 60 months after disease onset

At M60, the PCS and MCS scores were statistically significant between the recovered, affected, and CHIK- (P < .001). After separating the QoL in gender and current age groups, the PCS scores of female and male gender, and of individuals of age ≥ 30 years, and the MCS scores of females and individuals of age ≥ 60 years were significantly different between the 3 groups (P ≤ .001) (Table 5).

**Table 5:** QoL scores among cohort and non-cares, at 60 months after disease onset (n = 320)

	Recovered (R)		Affected (A)		CHIK- (NC)						
	(n = 107)		(n = 62)		(n = 151)			Difference		Difference	
	Median (IQR)	Mean (SD)	Median (IQR)	Mean (SD)	Median (IQR)	Mean (SD)	P-value^a^	(R – NC)	P-value^b^	(A – NC)	P-value^b^
**Physical functioning**	90.0 (60.0–100)	80.4 (26.0)	65.0 (43.8–90.0)	65.6 (28.4)	95.0 (80.0–100)	85.9 (27.0)	** < .001**	5.0	.27	− 30.0	** < .001**
**Social functioning**	100 (87.5–100)	90.2 (16.4)	87.5 (75.0–100)	84.5 (17.8)	100 (87.5–100)	92.6 (16.3)	** < .001**	0.0	.11	− 12.5	** < .001**
**Physical role functioning**	100 (100–100)	81.3 (36.0)	100 (0.0–100)	64.5 (44.0)	100 (100–100)	90.1 (27.9)	** < .001**	0.0	.03	0.0	** < .001**
**Emotional health perception**	100 (100–100)	89.7 (29.1)	100 (100–100)	83.3 (33.5)	100 (100–100)	94.1 (21.8)	.01	0.0	.24	0.0	.003
**Mental health**	92.0 (76.0–100)	85.5 (15.6)	84.0 (67.0–92.0)	79.5 (17.5)	92.0 (84.0–100)	87.6 (15.0)	.003	0.0	.46	− 8.0	** < .001**
**Vitality**	85.0 (65.0–95.0)	79.7 (17.3)	70.0 (55.0–85.0)	67.3 (20.5)	85.0 (75.0–95.0)	82.1 (15.1)	** < .001**	0.0	.39	− 15.0	** < .001**
**Bodily pain**	89.8 (67.4–100)	84.7 (19.7)	67.4 (48.5–79.6)	65.7 (20.6)	100 (79.6–100)	88.5 (18.4)	** < .001**	-10.2	.05	− 32.6	** < .001**
**General health perception**	75.0 (60.0–85.0)	71.7 (16.7)	55.0 (48.8–75.0)	58.3 (19.3)	75.0 (65.0–80.0)	74.4 (12.6)	** < .001**	0.0	.38	− 20.0	** < .001**
**PCS**^c^	87.4 (73.1–93.8)	79.5 (20.5)	66.2 (45.4–84.0)	63.5 (23.7)	91.3 (82.4–95.0)	84.7 (17.1)	** < .001**	-3.9	.05	− 25.1	** < .001**
**Gender**											
Female	89.7 (79.0–95.0)	82.4 (18.6)	64.0 (39.7–84.3)	61.3 (24.4)	88.8 (80.9–95.0)	84.2 (17.7)	** < .001**	0.9	.83	− 24.8	** < .001**
Male	83.8 (50.6–91.2)	73.2 (23.3)	80.9 (61.2–85.2)	75.1 (15.5)	92.5 (86.7–95.0)	85.7 (16.1)	** < .001**	-8.7	.002	-11.6	.004
**Age, years**											
23–29	96.3 (96.3–96.3)	96.3 (0.0)	81.5 (80.6–81.5)	81.5 (1.3)	92.5 (88.6–95.3)	92.7 (3.7)	.04	3.8	.18	− 11.0	.03
30–44	93.8 (82.5–95.9)	88.1 (12.4)	63.7 (46.8–82.4)	63.1 (20.5)	91.3 (86.8–96.3)	85.9 (18.1)	**.001**	2.5	.84	− 27.6	**.001**
45–59	89.3 (80.0–95.0)	84.3 (15.7)	77.1 (55.5–89.6)	70.1 (21.4)	91.9 (86.2–95.0)	89.2 (12.5)	** < .001**	− 2.6	.26	− 14.8	** < .001**
≥ 60	81.8 (47.8–91.2)	69.8 (24.3)	50.5 (29.0–78.4)	53.3 (26.0)	88.8 (77.7–93.8)	81.5 (18.9)	** < .001**	-7.0	.004	-38.3	** < .001**
**Comorbidities**											
Rheumatic disorders^d^	80.6 (44.0–89.2)	68.7 (24.7)	55.5 (31.9–66.5)	52.7 (19.7)	84.9 (59.3–88.6)	71.8 (23.9)	.01	− 4.3	.76	− 29.4	.006
Cardiovascular diseases^e^	82.4 (47.8–92.5)	73.1 (24.2)	63.1 (29.3–83.7)	58.0 (26.7)	82.4 (72.4–91.3)	76.0 (20.6)	.01	0.0	.94	− 19.3	.007
Diabetes mellitus	59.3 (37.8–90.3)	60.2 (27.9)	73.7 (23.2–85.6)	58.1 (31.4)	76.3 (62.1–88.7)	72.0 (22.4)	.43	− 17.0	.30	− 2.6	.29
Asthma	91.2 (75.9–96.3)	85.9 (12.4)	35.0 (26.2–77.1)	48.5 (25.1)	81.2 (34.4–92.5)	69.4 (33.1)	.02	10.0	.33	− 46.2	.33
Allergies^f^	92.4 (58.4–95.0)	78.5 (25.8)	79.0 (51.8–84.3)	69.5 (21.8)	92.5 (85.5–93.8)	85.5 (16.8)	.05	− 0.1	.71	− 13.5	.01
**MCS**^g^	92.1 (81.0–97.5)	86.5 (15.5)	85.7 (70.6–92.0)	78.6 (18.3)	93.3 (86.8–97.5)	89.1 (13.7)	** < .001**	− 1.2	.20	− 7.6	** < .001**
**Gender**											
Female	92.1 (81.4–98.8)	87.3 (14.3)	84.2 (65.3–90.3)	76.2 (18.7)	93.0 (86.3–96.4)	88.1 (14.6)	** < .001**	− 0.9	.99	− 8.8	** < .001**
Male	90.6 (78.7–96.5)	84.0 (18.0)	92.5 (89.9–96.6)	91.2 (7.7)	95.1 (88.5–98.8)	90.9 (11.9)	.07	− 4.5	.02	− 2.6	.41
**Age, years**											
18–29	99.5 (99.0–99.5)	99.5 (0.7)	88.6 (86.0–88.6)	88.6 (3.7)	90.9 (87.1–94.2)	89.6 (8.5)	.12	8.6	.06	− 2.3	.49
30–44	93.7 (85.0–99.7)	88.9 (13.6)	75.3 (64.4–87.0)	72.3 (20.0)	86.5 (73.4–90.3)	80.0 (18.9)	.01	7.2	.05	-11.2	.16
45–59	91.6 (82.5–97.5)	88.8 (11.3)	89.9 (72.1–94.0)	80.9 (19.1)	94.8 (90.9–98.8)	93.1 (8.0)	.003	− 3.2	.10	− 4.9	** < .001**
≥ 60	89.1 (75.7–96.6)	81.8 (19.2)	85.1 (66.4–90.2)	78.0 (16.9)	94.0 (86.8–97.3)	89.3 (14.2)	** < .001**	− 4.9	.03	− 8.9	** < .001**
**Comorbidities**											
Rheumatic disorders^d^	89.3 (78.3–97.6)	83.3 (18.0)	82.5 (57.4–86.6)	73.6 (18.1)	90.5 (71.3–95.0)	81.8 (18.2)	.05	− 1.2	.56	-8.0	.08
Cardiovascular diseases^e^	90.8 (77.6–98.1)	83.3 (18.7)	84.3 (69.4–90.8)	78.7 (15.4)	90.9 (85.1–94.1)	85.5 (15.9)	.06	-0.1	.93	-6.6	.01
Diabetes mellitus	81.6 (70.2–89.6)	76.5 (20.6)	89.1 (83.0–96.3)	84.2 (19.2)	92.8 (81.9–97.9)	85.1 (19.5)	.14	− 11.2	.07	− 3.7	.54
Asthma	94.0 (88.0–100)	93.2 (6.8)	82.5 (58.8–87.4)	75.0 (16.0)	88.4 (43.1–92.4)	74.6 (30.9)	.01	5.6	.11	− 5.9	.50
Allergies^f^	88.6 (64.7–98.8)	82.8 (18.2)	86.5 (74.4–92.3)	84.6 (10.5)	93.0 (70.9–95.3)	83.5 (19.7)	.87	− 4.4	.95	− 6.5	.48

^a^Two-sided P-value obtained using Kruskal–Wallis test; ^b^Two-sided P-value obtained using Mann-Withney U test as a post hoc test; ^c^Physical component summary (PCS) includes the domains Physical functioning, Physical role functioning, Bodily pain, and General health perception. ^d^Rheumatic disorders includes rheumatoid arthritis, joint pain, swelling, and weakness; ^e^Cardiovascular diseases includes, myocardial infarction, hypertension, hypotension, and hypercholesterolemia; ^f^Allergies includes hay fever, eczema, food intolerance and other. ^g^Mental component summary (MCS) includes the domains Social functioning, Emotional health perception, Mental health, and Vitality. SF-36 QoL scores from 0 (worst) to 100 (best). Significant P-values after Bonferroni correction are indicated in bold

At M60, a post hoc test was performed to evaluate the statistical significant difference between the PCS and MCS scores of the recovered and affected patients compared to the CHIK- (Table 5). The PCS and MCS scores of the recovered patients were not significantly different compared to the scores of the CHIK-. The PCS and MCS scores of the affected patients were negatively associated and significantly lower (P < .001) compared to the CHIK-. After separating the QoL scores in gender and current age, the PCS scores for female gender was significantly lower (P < .001) with a decrease of 24.8 points. The PCS scores were significantly lower (P .001) for age above 30 years with a decrease of points ranging from 14.8 to 38.3, when compared to the CHIK-. In addition, the MCS scores of the affected patients were significantly lower for female gender (P < .001) and individuals of 45–59 and ≥ 60 years, with a decrease of 4.9, 8.9, and 8.8 points, respectively.

## Discussion

To the best of our knowledge, the present longitudinal prospective study is the first to assess the long-term clinical manifestations and its impact on QoL, induced by infection with the Asian CHIKV genotype, using repeated assessments 60 months following disease onset, in the Caribbean region. Our primary findings at M60 include a high prevalence of persistent self-reported rheumatic symptoms of moderate to severe pain intensity, irrespective of age and underlying comorbidities, and a high prevalence of non-rheumatic symptoms in affected patients. Arthralgia in the upper and lower extremities, and headache were significantly associated with being long-term affected by chikungunya disease. In addition, at M60 the SF-36 PCS and MCS of the affected chikungunya patients were more impaired compared to the CHIK-, whom were not exposed to chikungunya disease.

Our findings add to the limited body of evidence that CHIKV infection can be associated with long-term clinical manifestations, 60 months after acute disease onset. The data reveal that more than one third of our study population self-declared being long-term affected by chikungunya disease, at M60. This is higher compared to a systematic review and meta-analysis from a total of 38 CHIKV studies conducted in 2018, which reported an overall non-recovery rate of 21%, between 12 and 24 months after disease onset [1]. However, the review did not exclude the possibility of CHIKV re-infection. In contrast to our study population, some study populations included in the review may have acquired protective long-term immunity from previous CHIKV outbreaks, and subsequently developed a decreased risk of long-term chikungunya disease during CHIKV re-infection [32, 33].

Following the long-term clinical symptoms 60 months after disease onset, the affected patients reported an increase of arthralgia in the lower extremities at M30 compared to M3-16, highlighting the recurrent nature of symptoms, which may be present or absent at time of the survey interviews, as have been discussed in our previous study [11]. At M60, all rheumatic symptoms were significantly more prevalent among the affected compared to the recovered patients. The main long-term clinical symptoms reported by the affected patients were rheumatic symptoms of recurrent nature, with more than 96% suffering from arthralgia, predominately in the upper and lower extremities. Moreover, almost half of the affected patients reported joint weakness in the upper and lower extremities, and 82.3% reported moderate to severe pain intensity.

In addition, although not the main focus of our study, our findings supports the results of a recent review [34], reporting that the prevalence of long-term arthralgia induced by the Asian CHIKV genotype is not lower compared to the IOL CHIKV genotype, as have been previously reported [1]. In contrast, even though a higher proportion of patients self-declared being affected by the IOL CHIKV genotype 5 years after disease onset, a lower arthralgia prevalence of 22% was reported, and yet a similar proportion of patients reported moderate to severe pain intensity compared to our study [35]. However, due to the disparities between the studies in terms of population characteristics including female/male proportion, prevalence of possible long-term immunity, and selection bias, more comparative studies are required to support these findings.

Moreover, in addition to rheumatic symptoms, affected patients suffered significantly higher from persistent non-rheumatic symptoms of recurrent nature, especially fatigue, insomnia, and psychological symptoms including loss of vitality, and headache compared to the recovered patients, at M60. Considering the aforementioned findings, long-term chikungunya disease assessment must include non-rheumatic/psychological symptoms sequelae alongside to persistent rheumatic symptoms, for the total burden of long-term chikungunya disease, as a IOL genotype study [20] and our previous study [11] reported that fatigue, psychological symptoms, and depressive moods may increase with time as chikungunya disease progresses.

The factors associated with persistent rheumatic symptoms after CHIKV are not well known. In our study population, age at acute disease onset nor increasing age during chikungunya disease sequelae, were found to be associated with persistent rheumatic symptoms among the affected patients, at M60. This finding, although contradicting several previous studies reporting age at the time of acute disease onset, especially those in the ≥ 35 [30] and ≥ 45 [27–29] age groups, to be predictive of persistent arthralgia and non-recovery after CHIKV infection, supports the results of other studies, which found a similar negative association between age with persistent rheumatic symptoms [36, 37]. Moreover, there were no significant differences in pre-existing and post-infection comorbidities including rheumatic disorders, CVD, DM, asthma, and allergies among the recovered and affected patients, as reported by others [36, 38]. Studies not excluding underlying comorbidities may promote over-estimation of disease severity due to wrongly attributing rheumatic symptoms to chikungunya disease [39]. Considering the aforementioned results, our study has shown that the persistent rheumatic symptoms is highly attributed to long-term chikungunya disease.

Arthralgia in the upper and lower extremities, and headache were significantly and independently associated with being long-term affected by chikungunya disease, at M60. Considering the years of functional impairment of the extremities and reported pain intensity after acute disease, it is of high importance to monitor joint involvement, as seen in RA [40]. Several chikungunya studies reported radiographic evidence of structural joint damage persistency and progression [39, 41], joint space narrowing and bone erosions in hands and feet in long-term affected patients [42]. Imaging methods could thus play a key role in documenting joint involvement, and identify patients that would potentially benefit from therapies effective in RA [43], who can be candidates for more frequent clinical follow-up assessments [12]. Although previous imaging findings are still contradicting, in the view of our results we strongly advocate comparative and repeated radiographs of the upper and lower extremities, to detect possible joint damage in affected patients in large-scale studies.

In addition, only the MCS QoL scores of the cohort improved over time. The PCS and MCS QoL scores of the recovered patients were less impaired compared to the affected patients following 60 months after disease onset. Over time, the MCS scores of the recovered patients improved significantly, while both the PCS and MCS scores of the affected patients remained the same. This finding may highlight the mental and emotional burden of persistent symptoms, causing the MCS scores of the recovered patients to improve, whereas the MCS score of those affected remained the same, since M3-16 [44].

Our results show that long-term chikungunya disease negatively impacts QoL, as reported by a range of IOL genotypes studies, ranging from 17 months to 6 years after acute disease [8, 19, 20, 44]. In addition, in line with these studies, at M60 recovered patients perceived their PCS and MCS QoL comparable to the CHIK-, irrespective of age and gender. The PCS and MCS of the affected patients were significantly impaired compared to the recovered patients and CHIK- [8, 19, 20]. In addition, the PCS and MCS QoL scores of the affected patients for female gender and individuals of age ≥ 30 years, was significantly more impaired compared to the CHIK-. Therefore, we postulate that our results may show that coping with additional moderate to severe pain due to persistent arthralgia next to the age-associated losses of physical functioning (for individuals in the age categories ≥ 45 years) or unexpected impairment of physical functioning due to young age (for those in the age category 30–44 years), may be psychological more challenging, resulting in a mentally debilitating experience, and lower MCS QoL in long-term affected patients.

Several limitations of this study need to be considered when interpreting the results. The patients included in this study, all presented with acute chikungunya disease at their general practitioners during the CHIKV outbreak. Patients with more severe clinical manifestation of chikungunya disease during acute chikungunya disease, might have been more likely to seek health-care, possibly introducing selection bias. Recall bias could have occurred particularly on pain intensity between the first and second follow-up surveys. In addition, the cohort was not genotyped for the infecting CHIKV genotype. However, all CHIKV genotype studies in Curaçao, the Caribbean, and the Americas during the outbreak time period belonged to the circulating Asian genotype [17, 18, 22], making it highly unlikely that the cohort was infected with a different CHIKV genotype. Moreover, similar to most previous longitudinal studies that aimed to evaluate long-term chikungunya disease manifestations, the cohort was not clinically examined by a clinician at baseline and follow-ups, to verify the self-declared symptoms. The use of questionnaires and the lack of objective physical assessment may have led to an overestimation of symptoms on the patient’s part. However, patient’s threshold for reporting symptoms like arthralgia and joint weakness may be lower compared to the threshold of the clinician for verifying a symptom like joint swelling, that may be visually present on physical examinations [34, 45]. In addition, the cohort was not tested for intercurrent dengue infection. Though, unlike CHIKV, dengue is not known to cause long-term musculoskeletal symptoms [46].

The response rate was low, due to the fact that many cohort patients moved to an unknown address and changed their phone numbers, between the first and second follow-up and could not be reached, affecting the retention rate. However, this may not have influenced the outcome of this study, due to the fact that moving is not a factor related to being recovered or affected by long-term chikungunya disease. Moreover, the study population in the current study is mostly composed of female patients. It has been suggested in a RA study that females report higher levels of pain and reduced physical functioning [47]. However, the population is representative of other studies with regard to gender, which reported that persistent chikungunya disease symptoms are more frequent in females than males [28]. Despite these limitations, the present study reports consistent evidence of a significant burden of long-term chikungunya disease manifestation.

In conclusion, our findings have broadened the perception and knowledge of the clinical manifestations of long-term chikungunya disease, induced by infection with the Asian CHIKV genotype. The study provides evidence that independent of CHIKV genotype, age at acute disease onset, and underlying comorbidities, severe long-term rheumatic symptoms may persist, 60 months after disease onset. Furthermore, infection with the Asian CHIKV genotype induce persistent long-term rheumatic and non-rheumatic symptoms, and QoL impairment comparable to the IOL genotype disease sequelae. Further research is needed to validate our findings, and to follow the long-term clinical manifestations and QoL impairment of not only the Asian and IOL CHIKV genotypes, but to draw further insight into the impact of each CHIKV genotype.

## Supplementary Information

**Additional file 1. **Overview of the study participants 60 months after disease onset (n=169).

**Additional file 2. **Characteristics cohort and chikungunya non-cases, 60 months after disease onset (n=320).

**Additional file 3. **Rheumatic and non-rheumatic symptoms reported by cohort, 3-16 and 30 months after disease onset (n=169).

**Additional file 4. **Nature of rheumatic and non-rheumatic symptoms reported by cohort, 60 months after disease onset (n=169).

**Additional file 5. **Persistent rheumatic symptoms of affected patients related to age at the time of interview (n=62).

**Additional file 6. **Pre-infection comorbidities of the affected patients in relation to persistent rheumatic symptoms (n=62).

**Additional file 7. **Post-infection comorbidities of the affected patients in relation to persistent rheumatic symptoms (n=62).

**Additional file 8. **The cohort SF-36 QoL scores over time (n=169).

**Additional file 9. **The SF-36 QoL scores among the recovered patients over time (n=107).

**Additional file 10. **The SF-36 QoL scores among the affected patients over time (n=62).

## Data Availability

The datasets generated and/or analysed during the current study are not publicly available due to participants did not consent to have their full provided information available for the public but are available from the corresponding author on reasonable request.

## Abbreviations

CHIKV: Chikungunya virus
ECSA: East Central South Africa
IOL: Indian Ocean Lineage
RA: Rheumatoid arthritis
QoL: Health-related quality of life
CVD: Cardiovascular diseases
DM: Diabetes mellitus
CHIK-: Chikungunya non-cases
VAS: Visual Analogue Scale
SF-36: 36-Item short-form health survey
PCS: Physical components summary
MCS: Mental component summary
SD: Standard deviations
IQR: Interquartile ranges
ORs: Odds ratios
CI: Confidence interval
