# Supplementary material for: The clinical manifestation and the influence of age and comorbidities on long-term chikungunya disease and health-related quality of life: a 60-month prospective cohort study in Curaçao
Source: BMC Infect Dis. 2022 Dec 16;22:948. doi: 10.1186/s12879-022-07922-1 (PMC9756924; doi:10.1186/s12879-022-07922-1)
Supplement: Supplementary file 1 — Additional file 1. Overview of the study participants 60 months after disease onset (n=169). [file 12879_2022_7922_MOESM1_ESM.docx]

**Additional file 1. Overview of the study participants 60 months after disease onset (n=169).**

|  | **n** |
| --- | --- |
| **Cohort participants with laboratory confirmation at baseline in 2015** | 304 |
| **Non-contacting reasons second follow-up survey in 2019** |  |
| Participant has died in 2017 | 4 |
| Participant not interviewed after contact, due to illness (Alzheimer) | 3 |
| Participant refused further participation first follow-up survey in 2017 | 20 |
| Participant has died between first and second follow-up surveys | 3 |
| Participant was not reached by phone nor house-visits after 3 failed attempts | 37 |
| Participant moved to foreign country | 1 |
| **Non-response reasons second follow-up survey in 2019** |  |
| Participant refused | 16 |
| Participant not interviewed after contact, due to circumstances | 37 |
| Participant not interviewed after contact, due to illness (Alzheimer) | 10 |
| Participant reached but not included in analyses due to loss of follow up at second follow-up  survey in 2017 | 4 |
| **Consenting cohort participants second follow-up survey in 2019** | **169** |
