# Supplementary material for: The clinical manifestation and the influence of age and comorbidities on long-term chikungunya disease and health-related quality of life: a 60-month prospective cohort study in Curaçao
Source: BMC Infect Dis. 2022 Dec 16;22:948. doi: 10.1186/s12879-022-07922-1 (PMC9756924; doi:10.1186/s12879-022-07922-1)
Supplement: Supplementary file 2 — Additional file 2. Characteristics cohort and chikungunya non-cases, 60 months after disease onset (n=320). [file 12879_2022_7922_MOESM2_ESM.docx]

**Additional file 2. Characteristics cohort and chikungunya non-cases, 60 months after disease onset (n=320).**

|  | **Total** | **Recovered** | **Affected** | **CHIK-** | **P-value^a^** |
| --- | --- | --- | --- | --- | --- |
| **Participants, no (%)** | 320 | 107 | 62 | 151 |  |
| **Gender, no (%)** |  |  |  |  | .02 |
| Female | 223 (69.7) | 74 (69.2) | 52 (83.9) | 97 (64.2) |  |
| Male | 97 (30.3) | 33 (30.8) | 10 (16.1) | 54 (35.8) |  |
| Sex ratio (male/female) | 0.43 | 0.45 | 0.19 | 0.56 |  |
| **Age current, years (%)** |  |  |  |  | .005 |
| 18-29 | 14 (4.4) | 2 (1.9) | 2 (3.2) | 10 (6.6) |  |
| 30-44 | 50 (15.6) | 20 (18.7) | 11 (17.7) | 19 (12.6) |  |
| 45-59 | 109 (34.1) | 43 (40.2) | 28 (45.2) | 38 (25.2) |  |
| ≥60 | 147 (45.9) | 42 (39.3) | 21 (33.9) | 84 (55.6) |  |
| **Education, no (%)** |  |  |  |  | .03 |
| Primary school or less | 68 (21.3) | 30 (28.0) | 14 (22.6) | 24 (15.9) |  |
| Secondary school | 59 (18.4) | 15 (14.0) | 9 (14.5) | 35 (23.2) |  |
| Intermediate vocational school | 124 (38.8) | 44 (41.1) | 29 (46.8) | 51 (33.8) |  |
| University (of applied sciences) | 69 (21.6) | 18 (16.8) | 10 (16.1) | 41 (27.2) |  |
| **Occupation, no (%)** |  |  |  |  | .006 |
| Unemployed/homemaker/student | 48 (15.0) | 15 (14.0) | 9 (14.5) | 24 (15.9) |  |
| Paid work | 177 (54.1) | 66 (61.7) | 41 (66.1) | 66 (43.7) |  |
| Retired | 99 (30.9) | 26 (24.3) | 12 (19.4) | 61 (40.4) |  |
| **Income, no (%)**^bc^ |  |  |  |  | .41 |
| 0-1000 ANG | 67 (23.7) | 19 (19.6) | 12 (21.8) | 36 (27.5) |  |
| 1001-3000 ANG | 124 (43.8) | 47 (48.5) | 23 (41.8) | 54 (41.2) |  |
| 3001-5000 ANG | 54 (19.1) | 14 (14.4) | 13 (23.6) | 27 (20.6) |  |
| ≥5000 ANG | 38 (13.4) | 17 (17.5) | 7 (12.7) | 14 (10.7) |  |
| **Comorbidities, no (%)** |  |  |  |  |  |
| Absence of comorbidities | 201 (62.8) | 58 (54.2) | 36 (58.1) | 107 (70.9) | .02 |
| Rheumatic disorders^d^ | 70 (21.9) | 34 (31.8) | 21 (33.9) | 15 (9.9) | **<.001** |
| Cardiovascular diseases^e^ | 107 (33.4) | 45 (42.1) | 25 (40.3) | 37 (24.5) | .005 |
| Diabetes mellitus | 41 (12.8) | 14 (13.1) | 10 (16.1) | 17 (11.3) | .57 |
| Asthma | 22 (6.9) | 9 (8.4) | 9 (14.5) | 4 (2.6) | .005 |
| Allergies^f^ | 39 (12.2) | 16 (15.0) | 10 (16.1) | 13 (8.6) | .16 |

^a^Groups were compared using the Fisher’s exact test, with Bonferroni multiple post hoc analysis, two-sided p-value corresponds to the comparison of the proportions between the recovered, affected, and non-cases groups; ^b^Antillian Guilder, 1 ANG = 0.55 United States; ^c^Total recovered group n = 97, total affected group n = 55, total CHIK- group n = 131; Dollar; ^d^Rheumatic disorders includes rheumatoid arthritis, joint pain, swelling, and weakness; ^e^Cardiovascular diseases includes, myocardial infarction, hypertension, hypotension, and hypercholesterolemia; ^f^Allergies includes hay fever, eczema, food intolerance and other. CHIK- = chikungunya non-cases. Significant P-values after Bonferroni correction are indicated in bold.
