# Supplementary material for: The clinical manifestation and the influence of age and comorbidities on long-term chikungunya disease and health-related quality of life: a 60-month prospective cohort study in Curaçao
Source: BMC Infect Dis. 2022 Dec 16;22:948. doi: 10.1186/s12879-022-07922-1 (PMC9756924; doi:10.1186/s12879-022-07922-1)
Supplement: Supplementary file 3 — Additional file 3. Rheumatic and non-rheumatic symptoms reported by cohort, 3-16 and 30 months after disease onset (n=169). [file 12879_2022_7922_MOESM3_ESM.docx]

**Additional file 3. Rheumatic and non-rheumatic symptoms reported by cohort, 3-16 and 30 months after disease onset (n=169).**

|  | **M3-16** | | | **M30** | | |
| --- | --- | --- | --- | --- | --- | --- |
|  | **Recovered** | **Affected** |  | **Recovered** | **Affected** |  |
|  | **n (%)** | **n (%)** | **P-value^a^** | **n (%)** | **n (%)** | **P-value^a^** |
| **Arthralgia** | 50 (46.7) | 47 (75.8) | **<.001** | 66 (61.7) | 54 (87.1) | **<.001** |
| **Arthralgia in the**^b^ |  | | | | | |
| back/neck | 19 (17.8) | 22 (35.5) | .015 | 28 (26.2) | 23 (37.1) | .165 |
| upper extremities^c^ | 35 (32.7) | 33 (53.2) | .010 | 36 (33.6) | 40 (64.5) | **<.001** |
| lower extremities^d^ | 35 (32.7) | 34 (54.8) | .006 | 50 (46.7) | 40 (64.5) | .037 |
| **Weakness in the^b^** |  | | | | | |
| back/neck | 16 (15.0) | 16 (25.8) | .103 | 13 (12.1) | 15 (24.2) | .054 |
| upper extremities^c^ | 26 (24.3) | 32 (51.6) | **<.001** | 17 (15.9) | 28 (45.2) | **<.001** |
| lower extremities^d^ | 23 (21.5) | 26 (41.9) | .008 | 21 (19.6) | 24 (38.7) | .011 |
| **Myalgia** | 26 (24.3) | 26 (41.9) | .024 | 35 (32.7) | 33 (53.2) | .010 |
| **Fatigue** | 25 (23.4) | 26 (41.9) | .015 | 33 (30.8) | 35 (56.5) | .002 |
| **Insomnia** | 25 (23.4) | 23 (37.1) | .076 | 25 (23.4) | 33 (53.2) | **<.001** |
| **Sombreness** | 8 (7.5) | 16 (25.8) | .002 | 14 (13.1) | 21 (33.9) | .003 |
| **Loss of vitality** | 14 (13.1) | 21 (33.9) | .003 | 17 (15.9) | 24 (38.7) | **.001** |
| **Numbness** | 10 (9.3) | 17 (27.4) | .004 | 15 (14.0) | 17 (27.4) | .042 |
| **Paraesthesia** | 9 (8.4) | 11 (17.7) | .086 | 7 (6.5) | 10 (16.1) | .062 |
| **Nausea** | 4 (3.7) | 9 (14.5) | .016 | 10 (9.3) | 13 (21.0) | .039 |
| **Vomiting** | 4 (3.7) | 1 (1.6) | .653 | 2 (1.9) | 4 (6.5) | .194 |
| **Abdominal pain^e^** | 5 (4.7) | 7 (11.3) | .128 | 7 (6.5) | 12 (19.4) | .021 |
| **Skin diseases** | 6 (5.6) | 4 (6.5) | 1.000 | 6 (5.6) | 14 (22.6) | .002 |
| **Alopecia** | 7 (6.5) | 11 (17.7) | .036 | 8 (7.5) | 15 (24.2) | .004 |
| **Headache^f^** |  |  |  | 25 (23.4) | 28 (45.2) | .006 |
| **Loss of appetite^f^** |  |  |  | 5 (4.7) | 13 (21.0) | .002 |
| **Sore throat^f^** |  |  |  | 11 (10.3) | 7 (11.3) | 1.000 |
| **Chills^f^** |  |  |  | 10 (9.3) | 15 (24.2) | .013 |
| **Sensitivity to light^f^** |  |  |  | 10 (9.3) | 18 (29.0) | .002 |

^a^Groups were compared using the Fisher’s exact test, with Bonferroni multiple post hoc analysis, two-sided p-value corresponds to the comparison of the proportions between the recovered and affected groups; ^b^Multiple answers possible; ^c^Upper extremities refers to the shoulders, elbows, hands, wrists, and fingers; ^d^Lower extremities refers to the hips, knees, ankles, feet, and toes. ^e^M3-16 total recovered group n = 106, total affected group n = 62; ^f^Non-rheumatic symptom measured since first follow-up survey, 30 months after disease onset. M3-16 = baseline survey: 3-16 months after disease onset; M30 = first follow-up survey: 30 months after disease onset. Significant P-values after Bonferroni correction are indicated in bold.
