# Supplementary material for: The clinical manifestation and the influence of age and comorbidities on long-term chikungunya disease and health-related quality of life: a 60-month prospective cohort study in Curaçao
Source: BMC Infect Dis. 2022 Dec 16;22:948. doi: 10.1186/s12879-022-07922-1 (PMC9756924; doi:10.1186/s12879-022-07922-1)
Supplement: Supplementary file 4 — Additional file 4. Nature of rheumatic and non-rheumatic symptoms reported by cohort, 60 months after disease onset (n=169). [file 12879_2022_7922_MOESM4_ESM.docx]

**Additional file 4. Nature of rheumatic and non-rheumatic symptoms reported by cohort, 60 months after disease onset (n=169).**

|  | **Recovered (n = 107)** | | **Affected (n = 64)** | |  |
| --- | --- | --- | --- | --- | --- |
|  | **Recurrent** | **Constant** | **Recurrent** | **Constant** | **P-value^a^** |
|  | **n (%)** | **n (%)** | **n (%)** | **n (%)** |  |
| **Arthralgia in the**^b^ |  |  |  |  |  |
| back/neck | 14 (63.6) | 8 (36.4) | 20 (64.5) | 11 (35.5) | 1.000 |
| upper extremities^c^ | 19 (90.5) | 2 (9.5) | 33 (71.7) | 13 (28.3) | .12 |
| lower extremities^d^ | 20 (69.0) | 9 (31.0) | 32 (61.5) | 20 (38.5) | .63 |
| **Weakness in the^b^** |  |  |  |  |  |
| back/neck | 4 (66.7) | 2 (33.3) | 11 (73.3) | 4 (26.7) | 1.000 |
| upper extremities^c^ | 7 (70.0) | 3 (30.0) | 20 (66.7) | 10 (33.3) | 1.000 |
| lower extremities^d^ | 7 (58.3) | 5 (41.7) | 16 (61.5) | 10 (38.5) | 1.000 |
| **Myalgia** | 14 (73.7) | 5 (26.3) | 28 (77.8) | 8 (22.2) | .75 |
| **Fatigue** | 14 (87.5) | 2 (12.5) | 16 (53.3) | 14 (46.7) | .03 |
| **Insomnia** | 7 (53.8) | 6 (46.2) | 13 (59.1) | 9 (40.9) | 1.000 |
| **Sombreness** | 7 (70.0) | 3 (30.0) | 15 (78.9) | 4 (21.1) | .67 |
| **Loss of vitality** | 10 (100) | 0 (0.0) | 20 (83.3) | 4 (16.7) | .30 |
| **Numbness** | 2 (100) | 0 (0.0) | 9 (81.8) | 2 (18.2) | 1.000 |
| **Paraesthesia** | 4 (80.0) | 1 (20.0) | 16 (76.2) | 5 (23.8) | 1.000 |
| **Nausea** | 3 (60.0) | 2 (40.0) | 11 (78.6) | 3 (21.4) | .57 |
| **Vomiting** | 0 (0.0) | 0 (0.0) | 2 (100) | 0 (0.0) | 1.000 |
| **Abdominal pain** | 1 (50.0) | 1 (50.0) | 2 (66.7) | 1 (33.3) | 1.000 |
| **Skin diseases** | 1 (33.3) | 2 (66.7) | 10 (71.4) | 4 (28.6) | .52 |
| **Alopecia** | 3 (100) | 0 (0.0) | 7 (46.2) | 7 (53.8) | .21 |
| **Headache^e^** | 10 (90.9) | 1 (9.1) | 23 (79.3) | 6 (20.7) | .65 |
| **Loss of appetite^e^** | 4 (100) | 0 (0.0) | 12 (85.7) | 2 (14.3) | 1.000 |
| **Sore throat^e^** | 1 (50.0) | 1 (50.0) | 4 (80.0) | 1 (20.0) | 1.000 |
| **Chills**^e^ | 2 (100) | 0 (0.0) | 9 (75.0) | 3 (25.0) | 1.000 |
| **Sensitivity to light^e^** | 2 (100) | 0 (0.0) | 5 (62.5) | 3 (37.5) | 1.000 |

^a^Groups were compared using the Fisher’s exact test, with Bonferroni multiple post hoc analysis, two-sided P-value corresponds to the comparison of the proportions of participants answering having ‘recurrent’ and those responding having ‘constant’ symptoms, between the recovered and affected groups; ^b^Multiple answers possible; ^c^Upper extremities refers to the shoulders, elbows, hands, wrists, and fingers; ^d^Lower extremities refers to the hips, knees, ankles, feet, and toes. ^e^Non-rheumatic symptom measured since the first follow-up survey 30, months after disease onset.
