# Supplementary material for: The clinical manifestation and the influence of age and comorbidities on long-term chikungunya disease and health-related quality of life: a 60-month prospective cohort study in Curaçao
Source: BMC Infect Dis. 2022 Dec 16;22:948. doi: 10.1186/s12879-022-07922-1 (PMC9756924; doi:10.1186/s12879-022-07922-1)
Supplement: Supplementary file 5 — Additional file 5. Persistent rheumatic symptoms of affected patients related to age at the time of interview (n=62). [file 12879_2022_7922_MOESM5_ESM.docx]

|  | **Current age categories (years)** | | | | | | | | |
| --- | --- | --- | --- | --- | --- | --- | --- | --- | --- |
|  | **Total** | **18-29** |  | **30-44** |  | **45-59** |  | **>60** |  |
|  | **(n = 62)** | **(n = 2)** |  | **(n = 11)** |  | **(n = 28)** |  | **(n = 21)** |  |
|  | **n (%)** | **n (%)** | **P-value**^a^ | **n (%)** | **P-value**^a^ | **n (%)** | **P-value**^a^ | **n (%)** | **P-value**^a^ |
| **Arthralgia in the**^b^ |  |  |  |  |  |  |  |  |  |
| back/neck | 31 (50.0) | 0 (0.0) | .49 | 6 (54.5) | 1.000 | 13 (46.4) | .80 | 12 (57.1) | .59 |
| upper extremities^c^ | 46 (74.2) | 2 (100) | 1.000 | 8 (72.7) | 1.000 | 22 (78.6) | .57 | 14 (66.7) | .37 |
| lower extremities^d^ | 52 (83.9) | 2 (100) | 1.000 | 10 (90.9) | .67 | 23 (82.1) | .74 | 17 (81.0) | .72 |
| **Weakness in the^b^** |  |  |  |  |  |  |  |  |  |
| back/neck | 15 (24.2) | 1 (50.0) | .43 | 3 (27.3) | 1.000 | 4 (14.3) | .14 | 7 (33.3) | .35 |
| upper extremities^c^ | 30 (48.4) | 1 (50.0) | 1.000 | 6 (54.5) | .75 | 11 (39.3) | .21 | 12 (57.1) | .42 |
| lower extremities^d^ | 26 (41.9) | 1 (50.0) | 1.000 | 6 (54.5) | .50 | 10 (34.6) | .20 | 10 (47.6) | .59 |
| **Myalgia** | 36 (58.1) | 2 (100) | .51 | 7 (63.6) | .75 | 13 (46.4) | .12 | 14 (66.7) | .42 |

**Additional file 5. Persistent rheumatic symptoms of affected patients related to age at the time of interview (n=62).**

^a^Groups were compared using the Fisher’s exact test, with Bonferroni multiple post hoc analysis, two-sided P-value corresponds to the comparison of the proportions of rheumatic symptoms and age categories among affected patients. ^b^Multiple answers possible; ^c^Upper extremities refers to the shoulders, elbows, hands, wrists, and fingers; ^d^Lower extremities refers to the hips, knees, ankles, feet, and toes.
