# Supplementary material for: The clinical manifestation and the influence of age and comorbidities on long-term chikungunya disease and health-related quality of life: a 60-month prospective cohort study in Curaçao
Source: BMC Infect Dis. 2022 Dec 16;22:948. doi: 10.1186/s12879-022-07922-1 (PMC9756924; doi:10.1186/s12879-022-07922-1)
Supplement: Supplementary file 6 — Additional file 6. Pre-infection comorbidities of the affected patients in relation to persistent rheumatic symptoms (n=62). [file 12879_2022_7922_MOESM6_ESM.docx]

**Additional file 6. Pre-infection comorbidities of the affected patients in relation to persistent rheumatic symptoms (n=62).**

|  | | **Pre-existing comorbidity, n (%)** | | | | | | | | | |
| --- | --- | --- | --- | --- | --- | --- | --- | --- | --- | --- | --- |
|  |  | **Rheumatic disorders^a^ n = 17 (27.4%)** | | **CVD^b^ n = 22 (35.5%)** | | **DM n = 10 (16.1%)** | | **Asthma n = 8 (12.9%)** | | **Allergies^c^ n = 8 (12.9%)** | |
|  | **Total** | **n (%)** | **P-value**^d^ | **n (%)** | **P-value**^d^ | **n (%)** | **P-value**^d^ | **n (%)** | **P-value**^d^ | **n (%)** | **P-value**^d^ |
| **Arthralgia in the^e^** |  |  |  |  |  |  |  |  |  |  |  |
| back/neck | 31 (50.0) | 11 (64.7) | .26 | 10 (45.5) | .79 | 6 (60.0) | .73 | 6 (75.0) | .26 | 3 (37.5) | .71 |
| upper extremities^f^ | 46 (74.2) | 16 (94.1) | .05 | 17 (77.3) | .77 | 6 (60.0) | .27 | 8 (100) | .10 | 7 (87.5) | .67 |
| lower extremities^g^ | 52 (83.9) | 16 (94.1) | .26 | 19 (86.4) | 1.000 | 8 (80.0) | .66 | 8 (100) | .33 | 6 (75.0) | .60 |
| **Weakness in the^e^** |  |  |  |  |  |  |  |  |  |  |  |
| back/neck | 15 (24.2) | 7 (41.2) | .09 | 5 (22.7) | 1.000 | 4 (40.0) | .24 | 5 (62.5) | .02 | 4 (50.0) | .09 |
| upper extremities^f^ | 30 (48.4) | 13 (76.5) | .01 | 13 (59.1) | .29 | 7 (70.0) | .18 | 6 (75.0) | .14 | 6 (75.0) | .14 |
| lower extremities^g^ | 26 (41.9) | 10 (58.8) | .15 | 9 (40.9) | 1.000 | 6 (60.0) | .30 | 7 (87.5) | .008 | 5 (62.5) | .27 |
| **Myalgia** | 36 (58.1) | 12 (70.6) | .26 | 11 (50.0) | .42 | 7 (70.0) | .50 | 8 (100) | .02 | 6 (75.0) | .45 |

^a^Rheumatic disorders includes rheumatoid arthritis, joint pain, swelling, and weakness; ^b^Cardiovascular diseases includes, myocardial infarction, hypertension, hypotension, and hypercholesterolemia; ^c^Allergies includes hay fever, eczema, food intolerance and other. ^d^Groups were compared using the Fisher’s exact test, with Bonferroni multiple post hoc analysis, two-sided P-value corresponds to the comparison of the proportions of rheumatic symptoms and comorbidities among affected patients. ^e^Multiple answers were possible; ^f^Upper extremities refers to the shoulders, elbows, hands, wrists, and fingers; ^g^Lower extremities refers to the hips, knees, ankles, feet, and toes. CVD = Cardiovascular diseases; DM = diabetes mellitus.
