# Supplementary material for: The clinical manifestation and the influence of age and comorbidities on long-term chikungunya disease and health-related quality of life: a 60-month prospective cohort study in Curaçao
Source: BMC Infect Dis. 2022 Dec 16;22:948. doi: 10.1186/s12879-022-07922-1 (PMC9756924; doi:10.1186/s12879-022-07922-1)
Supplement: Supplementary file 7 — Additional file 7. Post-infection comorbidities of the affected patients in relation to persistent rheumatic symptoms (n=62). [file 12879_2022_7922_MOESM7_ESM.docx]

**Additional file 7. Post-infection comorbidities of the affected patients in relation to persistent rheumatic symptoms (n=62).**

|  | | **Post-infection comorbidity, n (%)** | | | | | | | | | |
| --- | --- | --- | --- | --- | --- | --- | --- | --- | --- | --- | --- |
|  |  | **Rheumatic disorders**^a^ **n = 4 (6.5%)** | | **CVD**^b^ **n = 3 (4.8%)** | | **DM n = 0 (0.0%)** | | **Asthma n = 1 (3.2%)** | | **Allergies**^c^ **n = 2 (3.2%)** | |
|  | **Total** | **n (%)** | **P-value**^d^ | **n (%)** | **P-value**^d^ | **n (%)** | **P-value**^d^ | **n (%)** | **P-value**^d^ | **n (%)** | **P-value**^d^ |
| **Arthralgia in the**^e^ |  |  |  |  |  |  |  |  |  |  |  |
| back/neck | 31 (50.0) | 2 (50.0) | 1.000 | 1 (33.3) | 1.000 | 0 (0.0) | - | 1 (100) | 1.000 | 1 (50.0) | 1.000 |
| upper extremities^f^ | 46 (74.2) | 2 (50.0) | .27 | 2 (66.7) | 1.000 | 0 (0.0) | - | 1 (100) | 1.000 | 2 (100) | 1.000 |
| lower extremities^g^ | 54 (83.9) | 4 (100) | 1.000 | 2 (66.7) | .42 | 0 (0.0) | - | 1 (100) | 1.000 | 2 (100) | 1.000 |
| **Weakness in the**^e^ |  |  |  |  |  |  |  | 1 (100) |  |  |  |
| back/neck | 15 (24.2) | 0 (0.0) | .56 | 0 (0.0) | 1.000 | 0 (0.0) | - | 1 (100) | .24 | 1 (50.0) | .43 |
| upper extremities^f^ | 30 (48.4) | 2 (50.0) | 1.000 | 1 (33.3) | 1.000 | 0 (0.0) | - | 1 (100) | .48 | 1 (50.0) | 1.000 |
| lower extremities^g^ | 26 (41.9) | 2 (50.0) | 1.000 | 1 (33.3) | 1.000 | 0 (0.0) | - | 1 (100) | .42 | 1 (50.0) | 1.000 |
| **Myalgia** | 36 (58.1) | 2 (50.0) | 1.000 | 1 (33.3) | .57 | 0 (0.0) | - | 1 (100) | 1.000 | 2 (100) | .51 |

^a^Rheumatic disorders includes rheumatoid arthritis, joint pain, swelling, and weakness; ^b^Cardiovascular diseases includes, myocardial infarction, hypertension, hypotension, and hypercholesterolemia; ^c^Allergies includes hay fever, eczema, food intolerance and other. ^d^Groups were compared using the Fisher’s exact test, with Bonferroni multiple post hoc analysis, two-sided P-value corresponds to the comparison of the proportions of rheumatic symptoms and comorbidities among affected patients. ^e^Multiple answers possible; ^f^Upper extremities refers to the shoulders, elbows, hands, wrists, and fingers; ^g^Lower extremities refers to the hips, knees, ankles, feet, and toes. CVD = cardiovascular diseases; DM = diabetes mellitus.
