# Supplementary material for: The clinical manifestation and the influence of age and comorbidities on long-term chikungunya disease and health-related quality of life: a 60-month prospective cohort study in Curaçao
Source: BMC Infect Dis. 2022 Dec 16;22:948. doi: 10.1186/s12879-022-07922-1 (PMC9756924; doi:10.1186/s12879-022-07922-1)
Supplement: Supplementary file 8 — Additional file 8. The cohort SF-36 QoL scores over time (n=169). [file 12879_2022_7922_MOESM8_ESM.docx]

**Additional file 8. The cohort SF-36 QoL scores over time (n=169).**

| **Cohort (n = 169)** | | | | | | | | | | |
| --- | --- | --- | --- | --- | --- | --- | --- | --- | --- | --- |
|  | **M3-16** | | **M30** | | **M60** | |  |  |  |  |
|  | **Median (IQR)** | **Mean (SD)** | **Median (IQR)** | **Mean (SD)** | **Median (IQR)** | **Mean (SD)** | **P-value^a^** | **P-value^b^** | **P-value^c^** | **P-value^d^** |
| **Physical functioning** | 90.0 (60.0-100) | 76.4 (27.2) | 90.0 (55.0-100) | 76.5 (27.2) | 85.0 (55.0-100) | 75.0 (27.8) | .92 |  |  |  |
| **Social functioning** | 87.5 (75.0-100) | 83.1 (19.6) | 100 (75.0-100) | 87.4 (19.6) | 100 (75.0-100) | 88.1 (17.1) | **.001** | .04 | .004 | .52 |
| **Physical role functioning** | 100 (62.5-100) | 75.6 (39.5) | 100 (50.0-100) | 75.0 (40.6) | 100 (50.0-100) | 75.2 (39.9) | .91 |  |  |  |
| **Emotional health perception** | 100 (66.7-100) | 77.3 (39.9) | 100 (100-100) | 82.8 (35.5) | 100 (100-100) | 87.4 (30.8) | .007 |  |  |  |
| **Mental health** | 80.0 (70.0-88.0) | 77.1 (16.4) | 88.0 (76.0-100) | 84.4 (15.9) | 88.0 (72.0-96.0) | 83.3 (16.5) | **<.001** | **<.001** | **<.001** | .52 |
| **Vitality** | 70.0 (60.0-85.0) | 70.2 (18.5) | 75.0 (65.0-90.0) | 76.0 (18.4) | 80.0 (62.5-90.0) | 75.2 (19.4) | **<.001** | **<.001** | .003 | .42 |
| **Bodily pain** | 77.6 (57.1-100) | 73.7 (23.7) | 79.6 (67.4-100) | 77.7 (23.0) | 79.6 (67.4-100) | 77.7 (22.0) | .11 |  |  |  |
| **General health perception** | 70.0 (60.0-80.0) | 68.3 (17.9) | 75.0 (55.0-80.0) | 68.7 (18.4) | 70.0 (55.0-80.0) | 66.8 (18.8) | .47 |  |  |  |
| **PCS^d^** | 81.9 (62.4-90.3) | 73.5 (22.4) | 83.8 (59.3-92.5) | 74.5 (22.9) | 83.1 (55.8-92.4) | 73.7 (23.0) | .55 |  |  |  |
| **MCS^e^** | 84.8 (66.6-91.2) | 76.9 (19.6) | 90.3 (78.6-95.5) | 82.7 (19.1) | 89.3 (77.9-95.5) | 83.5 (17.0) | **<.001** | **<.001** | **<.001** | .64 |

^a^Two-sided P-value obtained using Friedman’s test; Two-sided P-value obtained using post hoc Wilcoxon test, comparing SF-36 QoL scores between ^b^baseline survey (3-16 months after disease onset) and first follow-up survey (30 months after disease onset), ^c^baseline and second follow-up survey (60 months after disease onset), and ^d^first follow-up survey and second follow-up survey. M3-16 = baseline survey: 3-16 months after disease onset; M30 = first follow-up survey: 30 months after disease onset; M60 = second follow-up survey: 60 months after disease onset. Physical component summary (PCS) includes the domains Physical functioning, Physical role functioning, Bodily pain, and General health perception; Mental component summary (MCS) includes the domains Social functioning, Emotional health perception, Mental health, and Vitality. SF-36 scores from 0 (worst) to 100 (best). Significant P-values after Bonferroni correction are indicated in bold.
