# Supplementary material for: The clinical manifestation and the influence of age and comorbidities on long-term chikungunya disease and health-related quality of life: a 60-month prospective cohort study in Curaçao
Source: BMC Infect Dis. 2022 Dec 16;22:948. doi: 10.1186/s12879-022-07922-1 (PMC9756924; doi:10.1186/s12879-022-07922-1)
Supplement: Supplementary file 9 — Additional file 9. The SF-36 QoL scores among the recovered patients over time (n=107). [file 12879_2022_7922_MOESM9_ESM.docx]

**Additional file 9. The SF-36 QoL scores among the recovered patients over time (n=107).**

|  | **M3-16** | | **M30** | | **M60** | |  |  |  |  |
| --- | --- | --- | --- | --- | --- | --- | --- | --- | --- | --- |
|  | **Median (IQR)** | **Mean (SD)** | **Median (IQR)** | **Mean (SD)** | **Median (IQR)** | **Mean (SD)** | **P-value^a^** | **P-value^b^** | **P-value^c^** | **P-value^d^** |
| **Physical functioning** | 90.0 (65.0-100) | 79.8 (26.3) | 95.0 (65.0-100) | 80.0 (25.6) | 90.0 (60.0-100) | 80.4 (26.0) | .808 |  |  |  |
| **Social functioning** | 87.5 (75.0-100) | 86.1 (17.4) | 100 (87.5-100) | 90.2 (16.6) | 100 (87.5-100) | 90.2 (16.4) | .003 |  |  |  |
| **Physical role functioning** | 100 (75.0-100) | 79.4 (37.3) | 100 (75.0-100) | 79.2 (38.0) | 100 (100-100) | 81.3 (36.0) | .552 |  |  |  |
| **Emotional health perception** | 100 (100-100) | 81.0 (37.2) | 100 (100-100) | 85.7 (33.4) | 100 (100-100) | 89.7 (29.1) | .116 |  |  |  |
| **Mental health** | 84.0 (72.0-92.0) | 79.4 (14.8) | 88.0 (80.0-100) | 85.1 (15.0) | 92.0 (76.0-100) | 85.5 (15.6) | **<.001** | **<.001** | **.001** | .696 |
| **Vitality** | 70.0 (60.0-85.0) | 72.0 (17.0) | 80.0 (70.0-95.0) | 78.6 (16.8) | 85.0 (65.0-95.0) | 79.7 (17.3) | **<.001** | **<.001** | **<.001** | .650 |
| **Bodily pain** | 79.6 (59.1-100) | 75.4 (22.6) | 89.8 (67.4-100) | 80.0 (22.8) | 89.8 (67.4-100) | 84.7 (19.7) | .002 |  |  |  |
| **General health perception** | 75.0 (60.0-85.0) | 71.3 (15.7) | 75.0 (60.0-85.0) | 70.7 (16.1) | 75.0 (60.0-85.0) | 71.7 (16.7) | .771 |  |  |  |
| **PCS** | 82.4 (70.0-91.3) | 76.5 (20.2) | 86.2 (69.2-93.8) | 77.5 (21.3) | 87.4 (73.1-93.8) | 79.5 (20.5) | .042 |  |  |  |
| **MCS** | 85.4 (72.3-91.8) | 79.6 (17.3) | 90.9 (82.4-96.5) | 84.9 (17.4) | 92.0 (80.9-97.5) | 86.3 (15.5) | **.001** | **.001** | **<.001** | .311 |

^a^Two-sided P-value obtained using Friedman’s test; Two-sided P-value obtained using post hoc Wilcoxon test comparing SF-36 QoL scores between ^b^baseline survey (3-16 months after disease onset) and first follow-up survey (30 months after disease onset), ^c^baseline and second follow-up survey (60 months after disease onset), and ^d^first follow-up survey and second follow-up survey. M3-16 = baseline survey: 3-16 months after disease onset; M30 = first follow-up survey: 30 months after disease onset; M60 = second follow-up survey: 60 months after disease onset. Physical component summary (PCS) includes the domains Physical functioning, Physical role functioning, Bodily pain, and General health perception; Mental component summary (MCS) includes the domains Social functioning, Emotional health perception, Mental health, and Vitality. SF-36 scores from 0 (worst) to 100 (best). Significant P-values after Bonferroni correction are indicated in bold.
