# Supplementary material for: The clinical manifestation and the influence of age and comorbidities on long-term chikungunya disease and health-related quality of life: a 60-month prospective cohort study in Curaçao
Source: BMC Infect Dis. 2022 Dec 16;22:948. doi: 10.1186/s12879-022-07922-1 (PMC9756924; doi:10.1186/s12879-022-07922-1)
Supplement: Supplementary file 10 — Additional file 10. The SF-36 QoL scores among the affected patients over time (n=62). [file 12879_2022_7922_MOESM10_ESM.docx]

**Additional file 10. The SF-36 QoL scores among the affected patients over time (n=62).**

|  | **M3-16** | | **M30** | | **M60** | |  |  |  |  |
| --- | --- | --- | --- | --- | --- | --- | --- | --- | --- | --- |
|  | **Median (IQR)** | **Mean (SD)** | **Median (IQR)** | **Mean (SD)** | **Median (IQR)** | **Mean (SD)** | **P-value^a^** | **P-value^b^** | **P-value^c^** | **P-value^d^** |
| **Physical functioning** | 80.0 (48.8-95.0) | 70.7 (28.1) | 80.0 (45.0-95.0) | 70.4 (28.9) | 65.0 (43.8-90.0) | 65.6 (28.4) | .585 |  |  |  |
| **Social functioning** | 87.5 (62.5-100) | 78.0 (22.3) | 100 (62.5-100) | 82.5 (23.2) | 87.5 (75.0-100) | 84.5 (17.8) | .232 |  |  |  |
| **Physical role functioning** | 100 (18.8-100) | 69.0 (42.6) | 100 (0.0-100) | 67.7 (44.2) | 100 (0.0-100) | 64.5 (44.0) | .442 |  |  |  |
| **Emotional health perception** | 100 (0.0-100) | 71.0 (43.7) | 100 (66.7-100) | 78.0 (38.6) | 100 (100-100) | 83.3 (33.5) | .062 |  |  |  |
| **Mental health** | 78.0 (64.0-84.0) | 73.0 (18.2) | 88.0 (75.0-100) | 83.2 (17.4) | 84.0 (67.0-92.0) | 79.5 (17.5) | **<.001** | **<.001** | .010 | .130 |
| **Vitality** | 65.0 (53.8-81.3) | 67.1 (20.7) | 75.0 (55.0-85.0) | 71.6 (20.2) | 70.0 (55.0-85.0) | 67.3 (20.5) | .053 |  |  |  |
| **Bodily pain** | 67.3 (53.1-89.8) | 70.7 (25.3) | 78.6 (67.4-92.4) | 73.7 (22.9) | 67.4 (48.5-79.6) | 65.7 (20.6) | .166 |  |  |  |
| **General health perception** | 70.0 (50.0-70.0) | 63.2 (20.2) | 70.0 (50.0-80.0) | 65.3 (21.5) | 55.0 (48.8-75.0) | 58.3 (19.3) | .035 |  |  |  |
| **PCS** | 77.8 (44.8-87.6) | 68.4 (25.1) | 79.2 (48.9-90.0) | 69.3 (24.7) | 66.2 (45.4-84.0) | 63.5 (23.7) | .195 |  |  |  |
| **MCS** | 81.4 (55.6-88.7) | 72.3 (22.4) | 86.8 (68.8-95.0) | 78.8 (21.4) | 85.7 (70.6-92.0) | 78.6 (18.3) | .015 |  |  |  |

^a^Two-sided P-value obtained using Friedman’s test; Two-sided P-value obtained using post hoc Wilcoxon test comparing SF-36 QoL scores between ^b^baseline survey (3-16 months after disease onset) and first follow-up survey (30 months after disease onset), ^c^baseline and second follow-up survey (60 months after disease onset), and ^d^first follow-up survey and second follow-up survey. M3-16 = baseline survey: 3-16 months after disease onset; M30 = first follow-up survey: 30 months after disease onset; M60 = second follow-up survey: 60 months after disease onset. Physical component summary (PCS) includes the domains Physical functioning, Physical role functioning, Bodily pain, and General health perception; Mental component summary (MCS) includes the domains Social functioning, Emotional health perception, Mental health, and Vitality. SF-36 scores from 0 (worst) to 100 (best). Significant P-values after Bonferroni correction are indicated in bold.
